# Molecular detection and characterization of *Anaplasma ovis*, *Theileria ovis*, and *Theileria lestoquardi* in sheep and goats in Luxor, Egypt

**DOI:** 10.1186/s12917-024-04109-5

**Published:** 2024-06-17

**Authors:** Hassan Y. A. H. Mahmoud, Tetsuya Tanaka, Alsagher O. Ali, Walaa F. A. Emeish

**Affiliations:** 1 Division of Infectious Diseases, Animal Medicine Department, Faculty of Veterinary Medicine, South Valley University, Qena, 83523 Egypt; 2 Laboratory of Infectious Diseases, Joint Faculty of Veterinary Medicine, Kagoshima University, Kagoshima, 890-0065 Japan; 3 Department of Fish Diseases and Management, Faculty of Veterinary Medicine, South Valley University, Qena, 83523 Egypt

**Keywords:** *Anaplasma ovis*, *Theileria ovis*, *Theileria lestoquardi*, Goat, Sheep

## Abstract

**Background:**

Tick-borne diseases cause economically significant losses to animal production globally, and anaplasmosis and theileriosis are associated with the greatest losses. However, the spread of the relevant pathogens in flocks of domesticated animals in southern Egypt is little understood. Accordingly, in this study, we aimed to determine the prevalences of *Anaplasma ovis*, *Theileria ovis*, and *Theileria lestoquardi* in southern Egyptian sheep and goats through blood tests, and to make a molecular characterization of the *A. ovis* detected in sheep targeting a specific gene.

**Results:**

We collected blood samples collected from 300 sheep and goats (*n*=150 /species) in Luxor Province in southern Egypt, and analyzed them for the presence of *A. ovis, T. ovis and T. lestoquardi* with screening by conventional and nested PCR targeting the *msp4* and *msp5, 18S rRNA,* and *merozoite surface protein* genes. For *A. ovis* 140/300 samples (46.66%) were positive overall, with 90/150 (60%) and 50/150 (33.33%) positive samples in sheep and goats, respectively. Two major surface protein genes of *A. ovis, msp4* and *msp5,* were sequenced using DNA extracted from sheep and goat blood samples, for phylogenetic analysis and genotyping. The *msp4* gene sequence revealed no significant genetic diversity, to contrast to data on *A. ovis* strains from other countries. For *T. lestoquardi*, 8/150 (5.33%) samples were positive in sheep, but no samples were positive in goats (0%). For *T. ovis,* 32/150 (21.33%) samples were positive in sheep, but no samples were positive in goats (0%). Sequencing targeting the *merozoite surface protein* gene for *T. lestoquardi* and the *small subunit ribosomal RNA* gene for *T. ovis* revealed no significant genetic diversity in the study, another contrast to data on *A. ovis* strains from other countries.

**Conclusion:**

This study provides valuable data on phylogenetic and molecular classifications of *A. ovis, T. ovis and T. lestoquardi* found in southern Egyptian sheep and goats. It also represents the first report on detection and molecular characterization of *T. lestoquardi* in southern Egyptian sheep based on the specific *merozoite surface protein* gene, thus providing valuable data for molecular characterization of this pathogen in southern Egypt.

## Introduction

Human and livestock health can be threatened by infectious diseases spread by vectors and wildlife [1]. Vector-borne diseases also cause significant economic losses due to high mortality rates and decreased productivity in livestock [2], and vectors spread around 25% of the pathogens that cause concerning diseases in vertebrates [3]. Numerous factors, including globalization and rising international trade, urbanization, climate change, travel, and animal migration, impact the epidemiology and spread of vector-borne diseases [4, 5].

Tick-borne diseases have global economic significance due to the costs associated with animal productivity losses, with anaplasmosis, babesiosis, and tropical theileriosis as diseases that cause some of the greatest economic losses [6]. *Anaplasma* organisms cause anaplasmosis and are Gram-negative obligate intracellular bacteria that infect multiple hosts. Anaplasmosis is a mild to severe intraerythrocytic disease that causes significant economic losses and changes in the condition of an infected animal rendering it more susceptible to potentially fatal bacterial, viral, or parasitic infections [7].

The most frequent cause of small ruminant anaplasmosis is *A. ovis*, which is found worldwide. The clinical symptoms of this condition include anorexia, decreased milk production, fever, tiredness, and abortion [8]. Anaplasmosis infection can occur together with other conditions, including stress, poor nutrition, tick infestation, hot weather, and co-infection with various bacteria or parasites. *A. ovis* infection can be more severe in goats than in sheep, especially in stressed or weak animals, and it is frequently asymptomatic [9]. *A. ovis* infection is reportedly relatively common and it is not necessarily linked with changes in any health-related variables [10].

Ruminants are important *Anaplasma* reservoir hosts, and this pathogen can cause persistent infection in animal hosts and cause the spread of the disease [11]. Traditional techniques for ascertaining *A. ovis* infection in animals include microscopic examination of Giemsa-stained blood smears; this process is not expensive, but it is time consuming, has low sensitivity, and its sensitivity depends on the examiner's skill level [12, 13]. In laboratory and field studies, *A. ovis* infection has been directly detected using alternative techniques such as PCR and loop-mediated isothermal amplification [14–16]. However, the sensitivity of these approaches is constrained, particularly in persistently infected animals, due to the low bacterial levels [17]. The use of serological assays to identify antibodies to *A. ovis* at all stages of infection has more advantages than other techniques [18].

Theileriosis is a serious hemoprotozoan disease that affects sheep and goats and is caused by pathogenic *Theileria* species, such as *Theileria luwenshuni*, *Theileria uilenbergi, and T. lestoquardi*. Non-pathogenic *Theileria* spp., such as *T.ovis*, *Theileria separata*, and *Theileria recondite*, frequently infect small ruminants [19–22]. Malignant theileriosis is caused by *T. lestoquardi*, which is found in tropical and subtropical areas of Asia, Africa, the Middle East, East and South Europe, and Africa [23, 24]. Following treatment, recovery from acute theileriosis infection is often accompanied by persistence of certain parasites, which is known as the carrier state. In this state, infection is frequently microscopically undetectable, but pathogen transmission may still occur through tick vectors [25, 26]. The presence of the vector in other theileriosis-free locations increases the probability of disease transmission significantly when an infected animal is in the carrier state. Significant reductions in cattle production have been associated with chronic asymptomatic *Theileria* spp carriage [27]. *T. ovis* is generally observed in sheep and goats, but it has also been found in water deer [28], dogs, dromedary camels [29, 30], and dairy cows [31]. *T. ovis* is non-pathogenic to goats and sheep [23], and there have been no clinical cases, so it may easily be overlooked.

Livestock, such as cattle, buffalo, and sheep, are the primary source of meat and milk for most of the population in Egypt, and a major source of income for farmers, accounting for 40% of the agricultural gross domestic product [32]. Anaplasmosis and theileriosis infecting sheep and goats in southern Egypt still have not been definitively profiled genetically, but such genetic profiling is essential for developing control measures against these diseases.

## Materials and methods

### Collection of samples and clinical examination

**Fig. 1:**
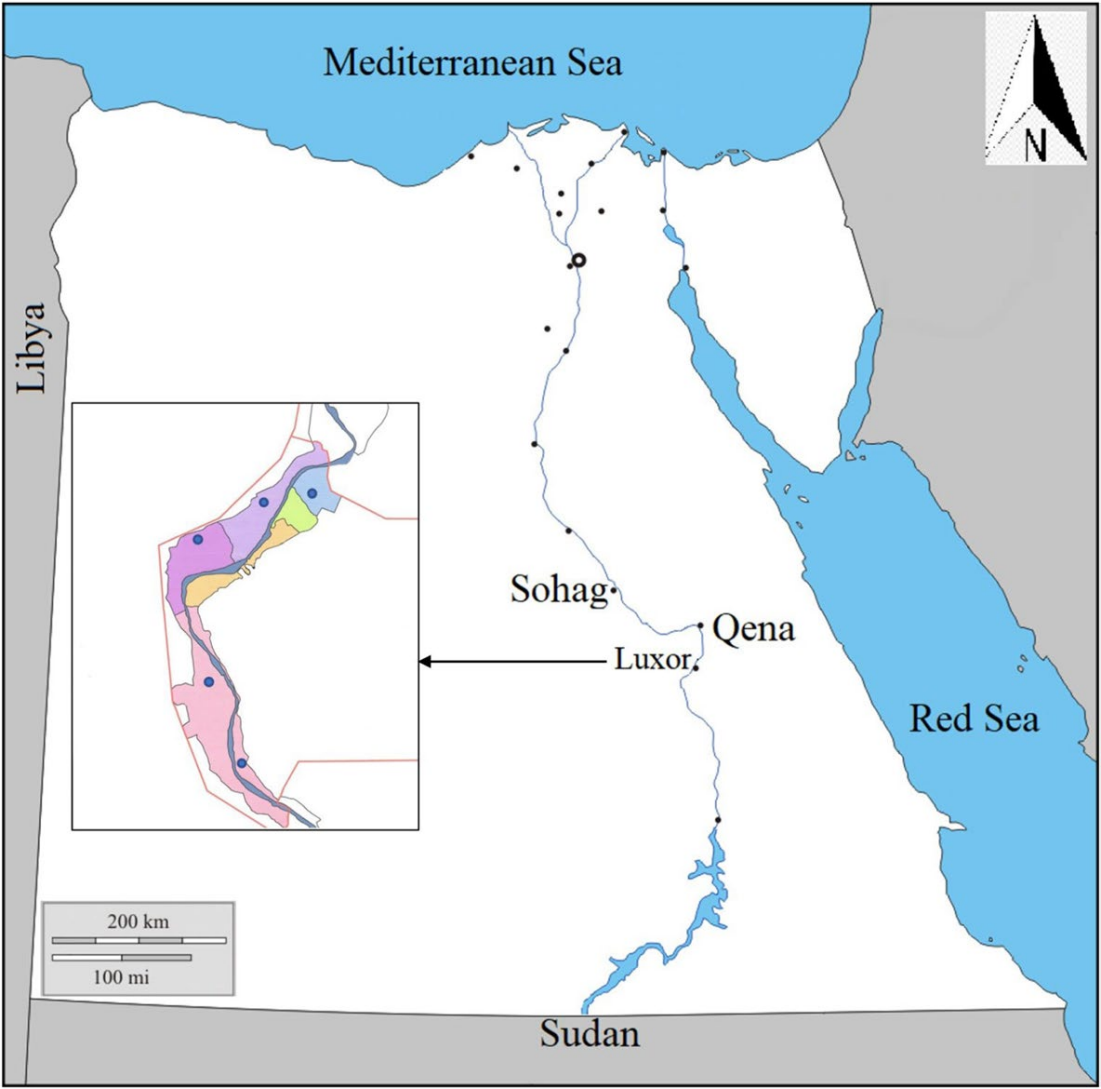
Map of Egypt indicating the study areas where samples were collected from different regions in Luxor

The current study focuses on anaplasmosis and theileriosis We targeted local breeds of sheep and goats of various ages and both sexes in the southern part of Egypt, in Luxor Province, for evaluation in the study with blood sample collection at the time of veterinary examinations between January 2021 to January 2022 (Fig.1). Each animal underwent a clinical evaluation before blood sample collection, and its age, sex, body mass index, body temperature, heart rate, respiratory rate, and visible mucous membrane condition were recorded [33].

A total of 300 animals were used in this study (150 sheep and 150 goats), of which 120 were male and 180 were female. The animals ranged in age from one to two years, and were classified into age groups (1-year-olds and 2-year-olds, with n=100 and n-200, respectively). Whole blood samples were taken from jugular vein of each animal using clean, sterile vacutainer tubes containing heparin for DNA extraction as a target for PCR amplification. DNA samples were kept at -20°C until analysis.

### Detection of pathogens by PCR

All primers used in this study are listed in Table 1 [34–37], and the PCR conditions are shown in Table 2. Negative controls containing nuclease-free water were used as negative samples. Electrophoresis of PCR products was performed with a 1.5% agarose gel in Tris–acetate–EDTA (TAE) buffer with a Mupid electrophoresis device (Mupid Co., Ltd., Tokyo, Japan), and bands were visualized through a gel documentation system UV device, WUV-M20 (ATTO Co., Ltd., Tokyo, Japan), after being stained with 5 μg /mL ethidium bromide in TAE buffer.

**Table 1:** The primers for detection of *Anaplasma ovis, Theileria ovis and Theileria lestoquardi* in sheep and goat

**Organism**	**Target gene**	**Primer Name**	**Sequence (5' → 3')**	**Expected size (bp)**	**References**
*Anaplasma marginale*	*msp5*	AM–49F	GTGTTCCTGGGGTACTCCTATGTGAACAAG	547	[34]
		AM–595R	AAGCATGTGACCGCTGACAAACTTAAACAG		
*Anaplasma ovis*	*msp4*	*msp45 F*	GGGAGCTCCTATGAATTACAGAGAATT GTT	854	[35]
		*msp43 R*	CCGGATCCTTAGCTGAACAGG AATCTTGC		
*Theileria ovis*	*18S rRNA*	*T.ovis F1*	TCGAGACCTTCGGGT	520	[36]
		*T.ovis R1*	TCCGGACATTGTAAAACAAA		
		*T.ovis F2*	CGCGTCTTCGGATGC	398	[36]
		*T.ovis R2*	AAAGACTCGTAAAGGAGCAA		
*Theileria lestoquardi*	*merozoite surface gene*	*T.les F*	GTGCCGCAAGTGAGTCA	785	[37]
		*T.les R*	GGACTGATGAGAAGACGATGAG

**Table 2:** PCR conditions for the amplification of target fragments of genes of *Anaplasma ovis, Theileria ovis and Theileria lestoquardi* in sheep and goat

**Target gene**	**PCR condition**
*msp4*	$\frac{{94}^{^\circ }\text{C}}{5\text{ min}}\to [ \frac{{94}^{^\circ }\text{C}}{30\text{ s}}-\frac{{60}^{^\circ }\text{C}}{30\text{ s}}-\frac{{68}^{^\circ }\text{C}}{1\text{min}} ] 35\times \to \frac{{68}^{^\circ }\text{C}}{7\text{ min}}\to {10}^{^\circ }\text{C}$∞
*msp5*^*a*^	$\frac{{95}^{^\circ }\text{C}}{5\text{ min}}\to [ \frac{{95}^{^\circ }\text{C}}{30\text{ S}}-\frac{{74-68}^{^\circ }\text{C}}{30\text{ S}}-\frac{{72}^{^\circ }\text{C}}{1\text{ min}} ] 36\times \to \frac{{72}^{^\circ }\text{C}}{5\text{ min}}\to {10}^{^\circ }\text{C}$∞
*Theileria ovis 18S rRNA*	$\frac{{95}^{^\circ }\text{C}}{5\text{ min}}\to [ \frac{{94}^{^\circ }\text{C}}{30\text{ S}}-\frac{{55}^{^\circ }\text{C}}{30\text{ S}}-\frac{{72}^{^\circ }\text{C}}{90\text{ S}} ] 35\times \to \frac{{72}^{^\circ }\text{C}}{7\text{ min}}\to {10}^{^\circ }\text{C}$∞
	$\frac{{95}^{^\circ }\text{C}}{3\text{ min}}\to [ \frac{{94}^{^\circ }\text{C}}{30 \text{S}}-\frac{{55}^{^\circ }\text{C}}{30\text{ S}}-\frac{{72}^{^\circ }\text{C}}{2\text{min}} ] 35\times \to \frac{{72}^{^\circ }\text{C}}{10\text{ min}}\to {10}^{^\circ }\text{C}$∞
*Theileria lestoquardi merozoite surface protein*	$\frac{{94}^{^\circ }\text{C}}{3\text{ min}}\to [ \frac{{94}^{^\circ }\text{C}}{1\text{ min}}-\frac{{55}^{^\circ }\text{C}}{1\text{ min}}-\frac{{72}^{^\circ }\text{C}}{1\text{min}} ] 35\times \to \frac{{72}^{^\circ }\text{C}}{7\text{ min}}\to {10}^{^\circ }\text{C}$∞

^a^Annealing with 0.2°C incremental decreases until reaching the final annealing temperature of 68°C

###  DNA extraction and PCR amplification

The 300 blood samples collected in this study (from 150 sheep and 150 goats), were subjected to DNA extraction (from whole blood) using commercial extraction kits (Wizard Genomic DNA Purification Kit, Promega, Madison, WI, USA). *A. ovis, T. ovis, and T. lestoquardi* were screened in a nested PCR targeting the *18S rRNA T. ovis* gene and conventional PCR targeting the *msp4* and *msp5,* and *merozoite surface protein* genes. The PCR reaction was performed with a total volume of 10 μL using Tks Gflex DNA Polymerase (TaKaRa, Shiga, Japan).

### Sequencing and data analysis

A subset of 50 samples was selected from the 300 study samples, and submitted to PCR assays for *A. ovis, T. ovis and T. lestoquardi* identification targeting the *msp4 and msp5, 18S rRNA and merozoite surface protein* genes (50 μL aliquots were used for sequence analysis). The amplicons were purified using a NucleoSpin Gel and PCR Clean-up kit (Macherey-Nagel, Leicestershire, Duren, Germany) following the manufacturer's protocol. Sequence readings were compared with sequences of reported isolates using BLAST in GenBank. A maximum likelihood phylogenetic tree was constructed using MEGA X software [38], with bootstrap values estimated using 1,000 replicates based on Kimura's two-parameter substitution model [39].

### Statistical analysis

The infection rates of *A. ovis, T. ovis and T. lestoquardi* in sheep and goats were determined by direct counting. Data were evaluated for significant differences and risk factors with a fisher exact probability test. The P value of < 0.05 was considered statistically significant. The 95% confidence intervals were calculated using Vassar Stats software (www.vassarstats.net.).

## Results

We detected *A. ovis* in 140/300 (46.66%) of samples overall, in 90/150 (60%) samples from sheep, and in 50/150 (33.33%) samples from goats (Table 3). *A. ovis* infection in sheep and goats was evaluated for statically significant associations with a number of risk factors (*p* < 0.05). By location, the highest prevalence of *A. ovis* infection was found in northern Luxor province (64 %), followed by the central region (46%) and southern Luxor provinces (40%). The location did not predict infection status when the northern and central regions were compared (*p* > 0.05); however, it showed significant prediction ability when the southern and northern regions were compared (*p* < 0.05). A higher infection rate (51%) was found in females than in males (40%), with a significant difference suggesting that sex was a risk factor (*p* < 0.05). The infection rate was higher in young animals (55%) than in older animals (42.5%). Assessing age as a risk factor, an age not exceeding one year showed a significant difference versus an age not exceeding two years (*p* < 0.05). Husbandry regime appears to play a role in the greater levels of *A. ovis* infection, with a lower rate for individual husbandry (37.5%) versus intensive husbandry (65%), and the significant difference suggested that husbandry was a risk factor (*p* < 0.05) (Table 4)

**Table 3:** Detection of *Anaplasma ovis, Theileria ovis and Theileria lestoquardi* infection rate in sheep and goat, from Luxor based on PCR detection in blood samples

Species	Number of animals	Number *of anaplasma ovis* positive	Percent *anaplasma ovis* positive	*Number of Theileria lestoquardi* positive	*Percent of Theileria lestoquardi positive*	Number of *Theileria ovis* positive	Percent of *Theileria ovis* positive
Sheep	150	90	60.00%	8	5.33%	32	21.33%
Goat	150	50	33.33%	0	0	0	0
Total	300	140	46.66 %	8	2.66%	32	10.66%

**Table 4:** *Anaplasma ovis* infection rate in sheep and goats in different locations in Luxor

**Factors**	**Locations**						**Age**				**Sex**				**Breeding system**			
	North		Middle		South		1 year		2 years		Male		Female		Individual		Intensive	
	N	%	N	%	N	%	N	%	N	%	N	%	N	%	N	%	N	%
Number of testing-positive animals	64	64	46	46	40	40	55	55	85	42.5	48	40	92	51	75	37.5	65	65
Number of testing-negative animals	36	36	54	54	60	60	45	45	115	57.5	72	60	88	49	125	62.5	35	35
Total number of animals tested	100		100		100		100		200		120		180		200		100	

*N* Number, *%* Percent

We detected *T. lestoquardi* in 8/150 (5.33%) of samples from sheep, but in no samples from goats (0/150; 0%).). Furthermore, we detected *T. ovis* in 32/150 (21.33%) of samples from sheep, but never detected in goats (Table 3). The goats in this study were negative for both *T. ovis* and *T. lestoquardi,* even though animals from both species in this study were gathered at the same location under both intensive and individual husbandry regimens*.*

In all *A. ovis*-positive samples, we detected major surface protein *msp4* and *msp5* genes, and they were sequenced for phylogenetic analysis and genotyping of this pathogen in the study samples from sheep and goats in Luxor Province in southern Egypt. All sequences were also submitted to GenBank, and the accession numbers shown below can be used to access them.

*msp4* gene: OP244840.1, OP244841.1, OP244842.1, and OP244843.1 in goats, and OP244844.1, OP244845.1, OP244846.1, and OP244847.1 in sheep

*msp5* gene: OP244852.1, OP244853.1, OP244854.1, and OP244855.1 in goats, and OP244848.1, OP244849.1, OP244850.1, and OP244851.1 in sheep

**Fig. 2:**
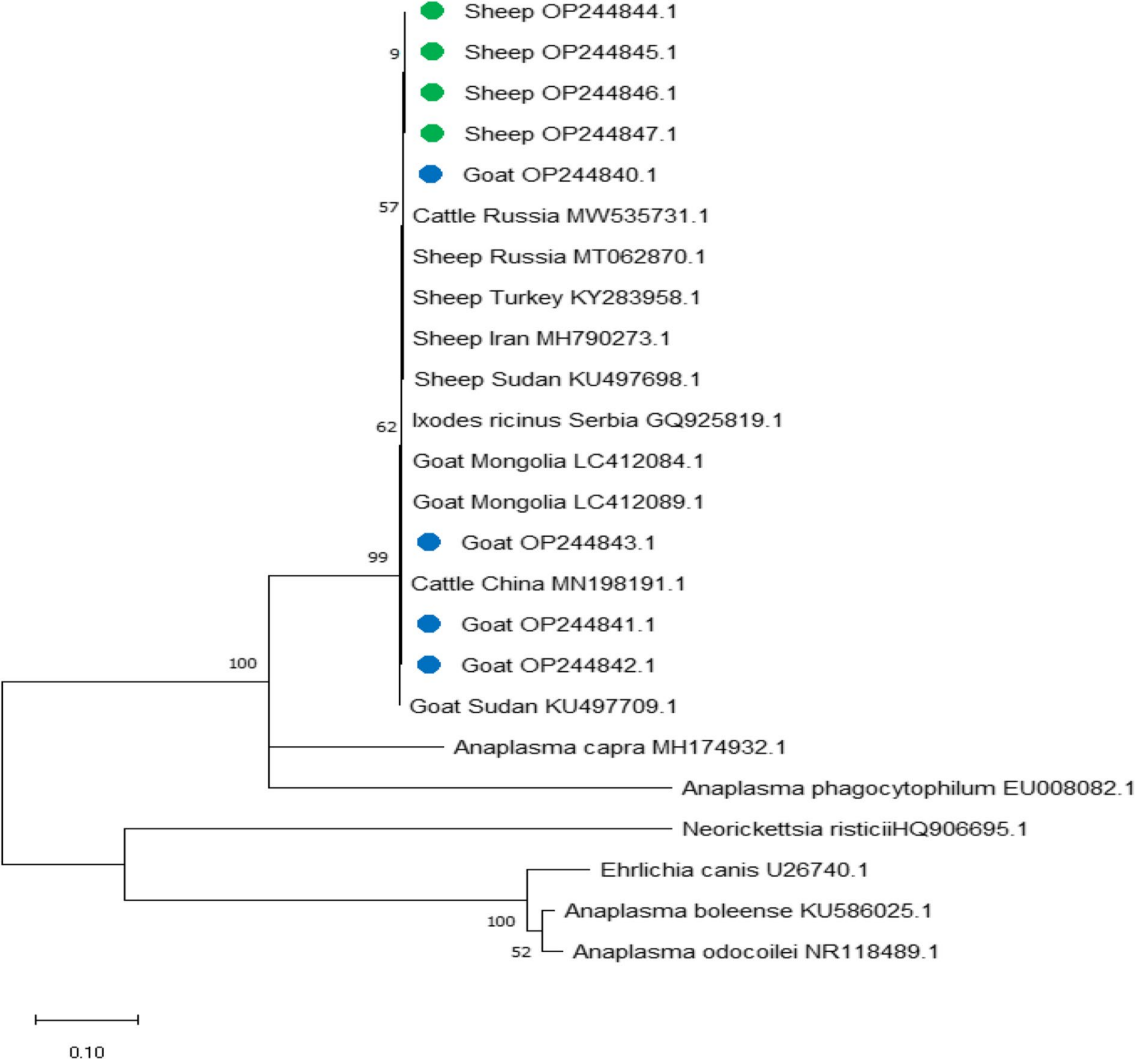
Phylogenetic relationships of *Anaplasma ovis* using the maximum likelihood method and Kimura’s 2-parameter model based on *major surface protein 4* (*msp4*) sequences. The percentage of trees in which the associated taxa clustered together is shown next to the branches. The tree is drawn to scale, with branch lengths measured in the number of substitutions per site. *Anaplasma ovis* obtained in the present study are represented by green and blue circles

**Fig. 3:**
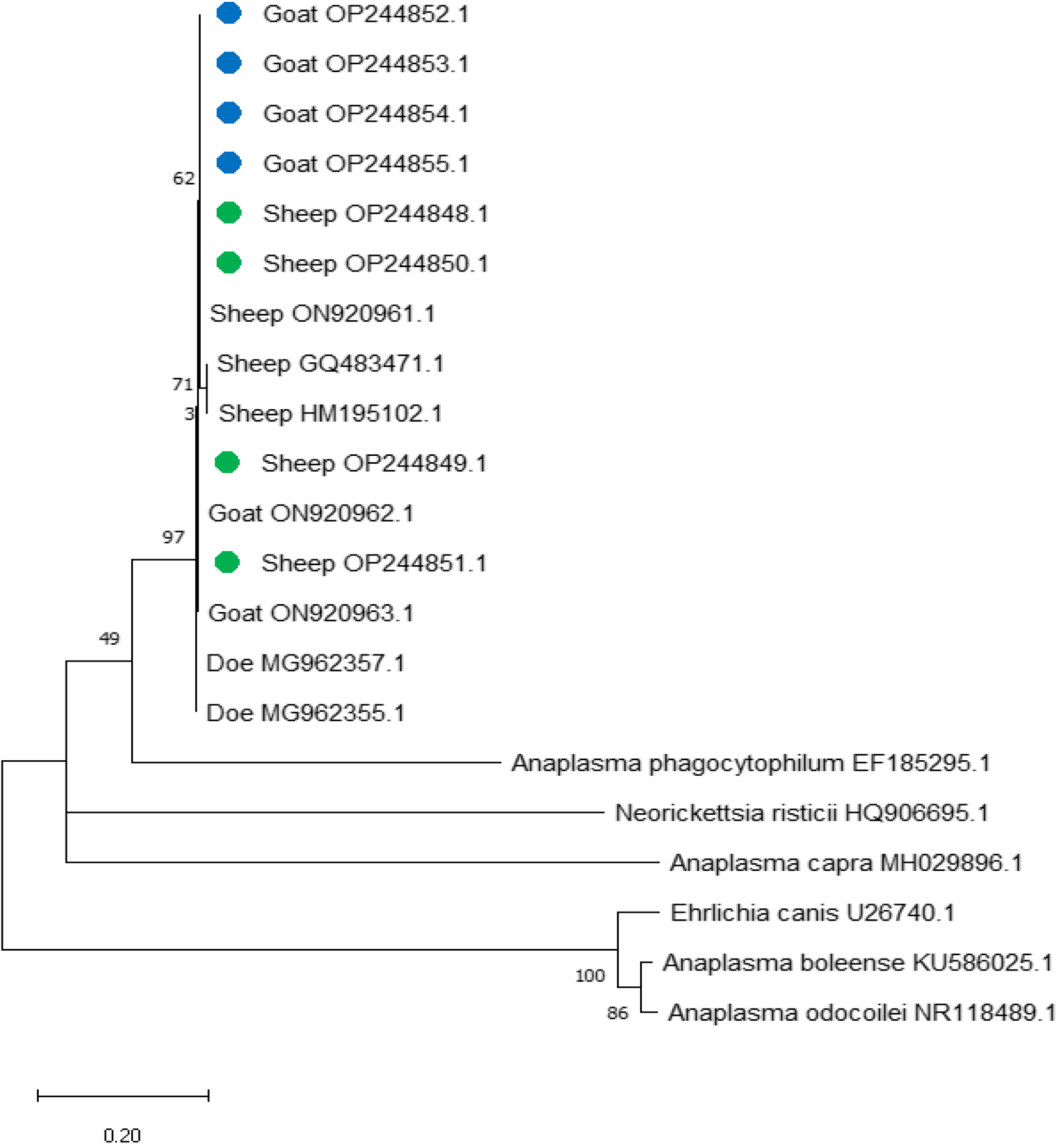
Phylogenetic relationships of *Anaplasma ovis* based on the *major surface protein 5* (*msp5*) sequences, the maximum likelihood method, and Kimura’s 2-parameter model. Next to the branches is a percentage of the trees in which the associated taxa clustered together. Branch lengths are expressed as the number of substitutions per site, and the tree is displayed to scale. Green and blue circles indicate the *Anaplasma ovis* results from the current study

The phylogenetic analysis for the *msp4* gene in sheep and goats in this study compared amplicons from other reported isolates, and alignment results demonstrated 100% identity to sheep from Iran (MH790273.1), Turkey (KY283958.1), and Russia (MT062870.1), and cattle from Russia (MW535731.1); 99.88% identity with sheep from Sudan (KU497698.1) and cattle from China (MN198191.1); 99.75% identity with goats from Mongolia LC412089.1 and LC412084.1; and 99.63% identity with goats from Sudan (KU497709.1), as well as with *Anaplasma* in *Ixodes ricinus* from Serbia GQ925819.1. The sequences for the *A. ovis* msp*4* gene were grouped with various isolates from other countries (Fig.2). The phylogenetic analysis for the *msp5* gene in sheep and goats in this study compared amplicons separate from other reported isolates with alignment by 99.81% identity to sheep from China GQ483471.1 and by 99.62% identity to sheep from China HM195102.1 (Fig.3).

**Fig. 4:**
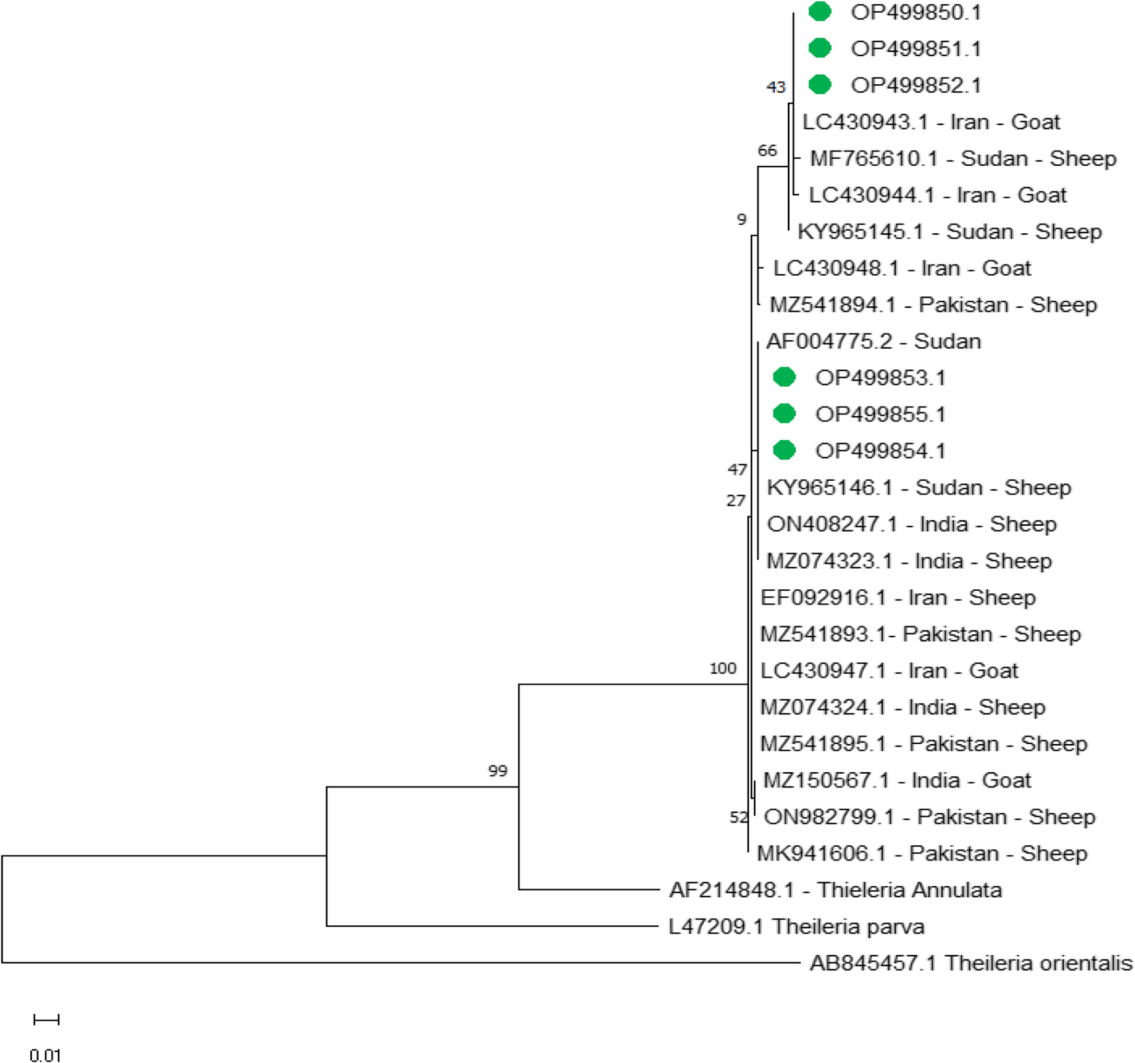
Phylogenetic relationships of *Theileria lestoquardi* using maximum likelihood method and Kimura 2-parameter model based on *merozoite surface protein*. The percentage of trees in which the associated taxa clustered together is shown next to the branches. The tree is drawn to scale, with branch lengths measured in the number of substitutions per site. *Theileria lestoquardi* obtained in the present study were represented by green circles

The *T. lestoquardi merozoite surface protein* sequences established for phylogenetic analysis and genotyping were classified into two groups. One group comprised OP499850.1, OP499851.1 and OP499852.1 and showed 100% identity to the LC430943.1 goat sequence from Iran deposited in GenBank sequences, and maximum identity (99.71%) with the LC430944.1 in sheep from Sudan, and minimum identity (97.16%) with the ON982799.1 in sheep from Pakistan. The second group comprised OP499853.1, OP499854.1, OP499855.1, and showed 100% identity with the sheep sequences from India, MZ074323.1, and ON408247.1, and with sheep sequences from Sudan, KY965146.1 and AF004775.2, but the minimum identity ( 99.04%) was with the MF765610.1 sequence from sheep in Sudan (Fig.4).

**Fig. 5:**
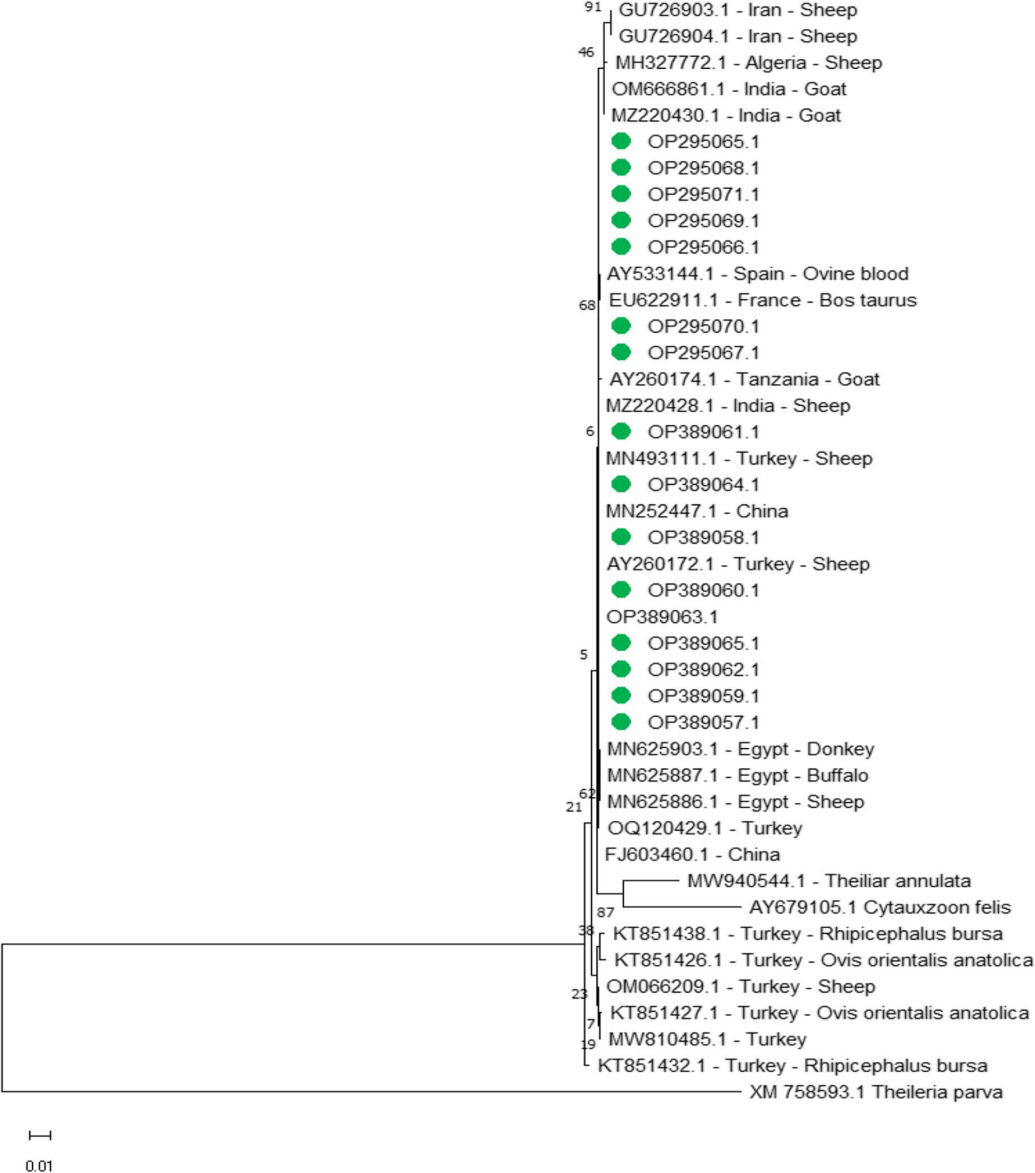
Phylogenetic relationships of *Theileria ovis* using maximum likelihood method and Kimura 2-parameter model based on *small subunit ribosomal RNA*. The percentage of trees in which the associated taxa clustered together is shown next to the branches. The tree is drawn to scale, with branch lengths measured in the number of substitutions per site. *Theileria ovis* obtained in the present study were represented by green circles

*T. ovis small subunit ribosomal RNA* was sequenced for phylogenetic analysis and genotyping, the result was submitted to GenBank with accession number from OP389057.1 to OP295071.1 the identical was by 100% with sequences from Egypt MN625903.1, MN625887.1 and MN625886.1 for donkey, boffola and sheep, and also identical by 100% with sheep from Turkey MN493111.1, sheep from Indian MZ220428.1 and sheep from Spain AY533144.1, in addition to it was identical by 99.88% with other sequences in GenBank with Sheep from Iran GU726904.1, Bos taurus from France EU622911.1 and goat from Indian MZ220430.1 (Fig.5).

## Discussion

The *major surface protein 5* is conserved across all known species of *Anaplasma marginale, Anaplasma centrale*, and *A. ovis* [40]. Without additional methods such as sequencing and multi-gene amplification, the diagnostic PCRs utilized for *A. ovis* and *A. marginale* identification have been unable to distinguish between these pathogens [41]. ELISA and PCR assays have been used to detect *A. ovis* in animals; however, these assays do not distinguish between different species, and a diagnosis of *A. ovis* infection is often made when screening samples from sheep [42, 43]. Assays targeting the species-specific *msp4* gene have been employed in a number of studies for epidemiological investigations and enabled researchers to clearly differentiate between *A. ovis* and *A. marginale* [44]. In North Africa, *A. ovis* infection has been studied in small ruminants in Egypt, Sudan, Tunisia, and Algeria [42, 45–47]. In the current study, the prevalence of *A. ovis* infection in sheep was 60%, lower than that reported in sheep in other North African countries, specifically, in Sudan at 60.1% [48], and Tunisa at 70.1% [49], and in Mongolia, at 69% [50]. However, our figure for prevalence in sheep exceeded those for Qinghai, China, at 54.5% [51], central and Western Kenya, at 34.2% [52], Turkey, at 29.7% [53], Iran, at 20.8% [54], Pakistan, at 12.5% [55], West Iran, at 10% [56], South Western China, at 5.7% [57], and North East China, at 2.6% [58].

In this study conducted in Egypt’s Luxor province, the rate of *A. ovis* infection in goats was 33.33%, which is higher than those detected by ELISA in goats in other Egyptian provinces, specifically in Alexandria (23.3%), Behirah (13.8%), Kafr El-Sheikh (22.6%), and El Gharbia (25%) [59].

In terms of age, we found a significant difference between one-year-old (age≤1) and two-year-old (age≤2 years) animals (*p* < 0.05). This result is consistent with the reportedly higher prevalence of *Anaplasma* spp infection in sheep below the age of six months [60], although it contrasts with previous reports that adult animals are more susceptible to infection [49, 54].

A higher infection rate (51%) was found in females than in males (40%). The significant difference indicated that sex was a predictive risk factor (*p* < 0.05), with females appearing more susceptible to *A. ovis* infection. This susceptibility may be explained by females experiencing greater hormonal fluctuations due to their reproductive cycle [49, 55].

In this study, we also suggested that location could be a predictive risk factor, with a significant difference between animals in the northern and southern regions of Luxor Province (*p* < 0.05), however, there was no significant difference between animals in the central and northern regions (*p* > 0.05).

Individual husbandry regime was associated with a lower infection rate (37.5%) than intensive husbandry (65%). The relevant risk factor showed a significant difference (p < 0.05); thus, husbandry may exert an effect whereby animals living in more intensive conditions are exposed to greater numbers of ticks [61]. The *msp4* gene sequence analysis in our study apparently did not reveal high genetic diversity for *A. ovis,* relative to results reported in other countries. The phylogenetic analysis based on this gene in the present study revealed 100% identity with sheep from Iran (MH790273.1), Turkey (KY283958.1), and Russia (MT062870.1), and cattle from Russia (MW535731.1), 99.88% identity with sheep from Sudan (KU4976981) and cattle from China (MN198191.1), 99.75% identity with Mongolian goats (LC412089.1 and LC412084.1), and 99.63% identity with Sudanese goats (KU497709.1), as well as *Ixodes ricinus* from Serbia GQ925819.1. Understanding the phylogenetic relationships between isolates of *A. ovis* is vital for informative analysis of intraspecific diversity that could contribute to better prevention and control of anaplasmosis. Therefore, sequencing of the *msp4* gene was used to analyze the diversity of our *A. ovis* samples.

The phylogenetic analysis of the *msp5* gene for sheep in this study did not show 100% identity with distinct amplicons from other reported isolates in GenBank. The highest figures were 99.81% identity with Chinese sheep (GQ483471.1) and 99.62% identity with another report in Chinese sheep (HM195102.1). The differences in our sequence were found in two sheep samples in one position, OP244849 and OP244851, in which nucleotide number 106 changed from G to A. This also led to a change in the amino acid from alanine to threonine. These changes are illustrated in Fig. 3 for both sequences in the phylogenetic tree for the *msp5* gene. These changes may be due to geographical or ecological factors, tick management, or control practices [16, 62]. Sheep and goats showed high *A. ovis* infection rates in this study. This may be the result of the limited diagnosis and treatment of sheep and goats with tick, and pasturing these animals in open areas in large numbers thus promoting the quick spread of infection between them. Regular surveys with good data are required to aid farmers and animal health practitioners, and provide guidance in controlling and prioritizing the health of sheep and goats in rural areas.

*Theileria lestoquardi* is a highly pathogenic ovine and caprine parasite that is believed to be the only *Theileria* species associated with economic loss present in small ruminants [63–65]. The parasite is spread by *Hyalomma anatolicum anatolicum*, which is found in South-eastern Europe, Northern Africa, Southern Russia, and the Middle East. However, *T. lestoquardi* distribution has been limited by the range of its vector.

*Theileria lestoquardi* was detected in a previous study in El-Wady El-Geded Governorate, Egypt, which targeted the *18S ribosomal RNA* gene for identification [66]. We consider that our study may be the first research in Egypt to characterize *T. lestoquardi* based on its *merozoite surface protein* gene for phylogenetic analysis, and to find 100% identity with reports from goats in Iran, and sheep in India and Sudan.

The results of this study was revealed that the total infection rate of *T. lestoquardi* was 8/150 (5.33%) in sheep which is higher than a previously reported figure in Egypt (0.87%) [66] but lower than that reported in Oman (10.6%) [67]. We consider that this is the first report on detection of *T. lestoquardi* based on the specific *merozoite surface protein*, and the epidemiology of *T. lestoquardi* needs further investigation in other parts of Egypt to fully elucidate its prevalence. We found a higher *T.ovis* infection rate, at 32/150 (21.33%), in sheep than previously reported in Egypt (4.37%) [66] and in Oman (2.7%) [67].

In this study, no goats in southern Egypt (Luxor prefecture) were infected with *T. lestoquardi* or *T.ovis*, an interesting finding that indicates the need for future research on piroplasmosis in this area. We speculate that *T. lestoquardi* causes subacute to acute theileriosis in indigenous sheep in the study area, in contrast to the inherent resistance or tolerance displayed by local sheep in other regions where *T. lestoquardi* is endemic, and that goats are more resistant to the infection than sheep. These variations may reflect parasite epidemiology, seasonal activity of vector ticks, the severity of tick infestation in hosts, behavioral differences between sheep and goats, and the small ruminant population in the studied location [68].

*T. ovis* primarily infects sheep and goats but has also been detected in water deer (Han et al. 2009), dogs, dromedary camels [29, 30], and dairy cows [31]. *T. ovis* is non-pathogenic in goats and sheep [23], with no clinical cases of infection having been observed in these species, and it is easily overlooked. *T. ovis* sequences obtained in the present investigation showed identity (100%) to *T. ovis* sequences detected in Egyptian donkeys, buffalos, and sheep, and have been grouped in the same clade as sequences of *T. ovis* from the various regions of Asia, Africa, and Europe. Moreover, these sequences showed strong similarities with corresponding sequences from sheep in the same clade from the Mediterranean area including Turkey, France, Spain, and Iran.

## Conclusions

This study focuses on anaplasmosis and theileriosis in local breeds of sheep and goats of various ages and both sexes, and demonstrated higher infection rates for *A. ovis* in animals under both intensive and individual husbandry regimens. This study represents the first report on the molecular characterization of *A. ovis* in goats in Egypt. Further research is required to assess the pathogenic potential of these diseases for sheep and goats and to elucidate the evolutionary changes and sources of variation, as information necessary for improvements in livestock health.

## Data Availability

The partial sequences of the *A. ovis msp4* and *msp5* genes have been deposited in GenBank with the accession numbers: OP244840.1, OP244841.1, OP244842.1, OP244843.1, OP244844.1, OP244845.1, OP244846.1, OP244847.1, OP244852.1, OP244853.1, OP244854.1, OP244855.1, OP244848.1, OP244849.1, OP244850.1, and OP244851.1. The partial sequences of the *T. lestoquardi merozoite surface protein* have been deposited in GenBank with the accession numbers: OP499850.1, OP499851.1, OP499852.1, OP499853.1, OP499854.1, and OP499855.1. The partial sequences of *T. ovis small subunit ribosomal RNA* deposited in GenBank with the accession numbers from OP389057.1 to OP295071.1.
